# The Vagal Link: Autonomic Nervous System Dysfunction in Cardiac Amyloidosis

**DOI:** 10.3390/jcm14248963

**Published:** 2025-12-18

**Authors:** Federico Barocelli, Eleonora Canu, Nicolò Pasini, Isabella Allegri, Alessandro D’Orsi, Alberto Bettella, Antonio Crocamo, Filippo Luca Gurgoglione, Laura Torlai Triglia, Francesca Russo, Angela Guidorossi, Maria Francesca Notarangelo, Nicola Gaibazzi, Livia Ruffini, Giampaolo Niccoli

**Affiliations:** 1 Cardiology Division, Parma University Hospital, 43126 Parma, Italy; fbarocelli@ao.pr.it (F.B.); eleonora.canu@unipr.it (E.C.); nicolo.pasini@unipr.it (N.P.); alberto.bettella@unipr.it (A.B.); acrocamo@ao.pr.it (A.C.); filippolucagurgoglione@gmail.com (F.L.G.); laura.torlait@gmail.com (L.T.T.); russofr@ao.pr.it (F.R.); aguidorossi@ao.pr.it (A.G.); notarangelof@ao.pr.it (M.F.N.); ngaibazzi@ao.pr.it (N.G.); 2 Neurology Division, Parma University Hospital, 43126 Parma, Italy; iallegri@ao.pr.it (I.A.); adorsi@ao.pr.it (A.D.); 3 Nuclear Medicine Unit, University Hospital of Parma, 43126 Parma, Italy; lruffini@ao.pr.it

**Keywords:** cardiac amyloidosis, autonomic dysfunction, transthyretin amyloidosis, amyloid neuropathy, vagus nerve, autonomic nervous system, heart-brain axis, orthostatic hypotension

## Abstract

Cardiac amyloidosis (CA) is an increasingly recognized cause of restrictive cardiomyopathy in which extracellular amyloid deposition leads to progressive structural and functional impairment. Beyond myocardial infiltration, growing evidence highlights the central role of autonomic nervous system dysfunction (ANS) —particularly the vagal nerve involvement—as a contributor to orthostatic intolerance, syncope, exercise limitation, arrhythmias, and reduced quality of life. Emerging data suggest that autonomic impairment may precede overt cardiac manifestations, offering a potential window for earlier recognition. This narrative review summarizes current knowledge on the mechanisms and clinical relevance of autonomic dysfunction in CA, emphasizing the novelty of the “vagal link” as a unifying framework connecting with a specific focus on the vagus nerve (VN) and its complex interplay with cardiac structure and function. We further discuss diagnostic approaches and the potential role of autonomic assessment in early detection, risk stratification, and personalized treatment strategies. A clearer understanding of vagal dysfunction may provide new insights into disease progression and identify opportunities for therapeutic innovation.

## 1. Introduction

Amyloidosis comprises a heterogeneous group of protein misfolding disorders characterized by the extracellular deposition of insoluble fibrillar aggregates that disrupt normal tissue architecture and function. More than thirty precursor proteins have been identified in humans, but systemic disease in adults is mainly caused by immunoglobulin light chains (AL amyloidosis) or transthyretin (ATTR amyloidosis) [1,2,3].

In ATTR, disease expression arises either from age-related loss of protein homeostasis in the wild-type form (ATTRwt) or from the presence of pathogenic variants in the TTR gene that destabilize the transthyretin protein in the hereditary form (ATTRv). Clinically, ATTRwt manifests primarily as a restrictive cardiomyopathy—transthyretin cardiac amyloidosis (ATTR-CA)—whereas ATTRv more commonly presents with a mixed phenotype that includes both cardiomyopathy and peripheral or autonomic neuropathy, showing variable phenotypic expression partly depending on the individual patient’s pathogenic TTR variant [2].

Cardiac amyloidosis (CA) has traditionally been considered a rare and underdiagnosed condition; however, recent epidemiologic and imaging-based screening studies indicate that ATTR-CA may be present in up to 13–15% of older individuals with Heart Failure with Preserved Ejection Fraction (HFpEF) and in 5–10% of those with severe aortic stenosis undergoing transcatheter valve replacement [4,5]. Although AL amyloidosis remains less common, its cardiac involvement carries a particularly aggressive course and accounts for the majority of early deaths among affected patients [1,3].

Among the extracardiac features of CA, autonomic nervous system (ANS) dysfunction represents one of the most distinctive yet underrecognized manifestations. Dysautonomia is particularly prominent in ATTRv, where it may precede overt cardiomyopathy or peripheral neuropathy by several years [6,7]. In contrast, autonomic involvement in AL amyloidosis is variable, while in ATTRwt it is often subclinical or mild [8]. The presence of autonomic dysfunction (AD) has prognostic implications, as it contributes to a variety of clinical manifestations that significantly affect quality of life and clinical outcomes [9].

The “vagal link”—the functional and structural interplay between the vagus nerve (VN) and the heart—has emerged as a conceptual framework for understanding the autonomic component of CA. The VN represents the principal parasympathetic conduit to the heart, modulating heart rate, atrioventricular conduction, and anti-inflammatory signaling through the cholinergic reflex arc [10]. Amyloid infiltration of autonomic ganglia, parasympathetic fibers, or the cardiac conduction system may therefore disrupt this regulatory axis, leading to a characteristic shift toward sympathetic predominance and baroreflex failure [10,11].

Despite increasing recognition of these findings, the role of AD in CA has not been systematically integrated into the diagnostic or prognostic paradigm. To our knowledge, most literature has traditionally focused on structural or hemodynamic cardiac parameters, whereas neural mechanisms have received comparatively little attention. Moreover, the relationship between autonomic imbalance and amyloid burden, conduction abnormalities, or arrhythmia risk remains incompletely defined.

This narrative review aims to provide an updated, integrative overview of ANS dysfunction in CA, with particular emphasis on the vagal link. We will explore the physiology of vagal–cardiac regulation, the pathophysiological mechanisms underlying autonomic impairment in amyloidosis, the clinical spectrum of dysautonomia across amyloid subtypes, and comparative data from other infiltrative or metabolic cardiomyopathies. By clarifying the neural dimension of CA, we aim to highlight its diagnostic and prognostic significance and to stimulate further investigation into the cardio-neural mechanisms underlying this complex disorder.

## 2. The Autonomic Nervous System and the Vagus Nerve: A Cardio-Neural Perspective

The ANS maintains cardiovascular homeostasis through a dynamic interplay between sympathetic adrenergic drive and parasympathetic (vagal) cholinergic restraint, whose net interaction defines the sympathovagal balance.

Vagal efferents originating from medullary nuclei project their preganglionic fibers to the cardiac plexus and intrinsic ganglia distributed mainly around the sinoatrial and atrioventricular nodes. These plexi, primarily located around the sinoatrial and atrioventricular nodes, exert negative chronotropic and dromotropic effects that stabilize rhythm and oppose excessive sympathetic excitation [12,13].

In parallel, cardiopulmonary and vascular afferents conveyed via the VN continuously update central autonomic outflow, coupling pressure/volume sensing with moment-to-moment adjustments of heart rate and vascular tone [14]. Functional integrity of the VN can be assessed through HRV, baroreflex sensitivity (BRS), and cardiovagal reflex testing (e.g., deep breathing or Valsalva ratio).

The frequency pattern of HRV provides a robust window on neurocardiac control at the sinus node [15,16]. Reduction in these indices indicates diminished vagal tone and is associated with increased risk of arrhythmia, HF progression, and mortality in diverse cardiac diseases [14].

Cardiac regulation is not executed solely by extrinsic autonomic fibers. An intrinsic neural network—the intrinsic cardiac nervous system (ICNS)—provides local integration of afferent inputs and efferent outputs and modulates nodal automaticity and atrial electrophysiology in close coordination with extrinsic vagal and sympathetic projections [13,14,15,16,17,18,19]. In practical terms, the integration of these systems together determines the phenotype of neurocardiac control recorded by heart rate variability/baroreflex sensitivity and observed at the bedside.

Within this framework, Porges [20] and Hayano et al. [16] further emphasize that higher cardiac vagal tone reflects greater physiological flexibility and resilience to perturbations, whereas vagal withdrawal denotes vulnerability to arrhythmogenesis and adverse outcomes.

In summary, this dynamic equilibrium regulates chronotropy, inotropy, and vascular tone, ensuring rapid adaptation to physiological demands such as orthostatic stress or exercise [12].

Beyond these hemodynamic regulatory features, the VN also exerts anti-inflammatory and anti-apoptotic influences through the cholinergic anti-inflammatory pathway (CAP). In fact, from the neuroimmune interface point of view, reduced HRV associates with higher circulating inflammatory mediators [e.g., Interleukin 6 (IL-6); Tumor Necrosis Factor Alpha (TNF-α)], supporting a functional linkage between vagal activity and systemic inflammatory burden [21,22]. Conceptually, HRV thus operates as an integrated biomarker of both neurocardiac regulation and inflammatory tone [22,23].

In cardiovascular disease models, augmenting vagal efferent activity attenuates cytokine responses, reduces oxidative/inflammatory stress, highlighting clinically relevant anti-inflammatory and antiarrhythmic properties of vagal modulation [24,25]. Contemporary perspectives underscore the parasympathetic–sympathetic crosstalk at reticuloendothelial sites, which further integrates these immunoregulatory effects [26].

The pathophysiological implications are direct: when cardiovagal integrity declines—through neuropathy or disease-related remodeling—the loss of parasympathetic restraint promotes sympathetic predominance, reduces HRV, impairs BRS, and dismantles the CAP. This could predispose to arrhythmias, orthostatic/hemodynamic lability, and a pro-inflammatory state [12,21,22,24,26].

Current evidence indicates that autonomic reflex impairment can be detected even before overt structural disease in ATTRv, emphasizing that cardiovagal dysfunction may be an early and measurable feature of the disease continuum [27].

Early electrophysiological studies corroborate reduced vagal control of conduction in hereditary amyloidosis [28].

These convergent lines of evidence establish the vagal link—a conceptual bridge connecting autonomic balance, intrinsic cardiac neural integration, and inflammatory restraint.

## 3. Pathophysiology of Autonomic Dysfunction in Cardiac Amyloidosis

AD represents a key, but often underrecognized, component of CA [29,30,31], contributing to hemodynamic instability, arrhythmic vulnerability [32,33,34], and poor prognosis [8,35,36,37]. Its pathophysiology reflects a multifactorial process encompassing direct amyloid infiltration of autonomic structures, neurodegeneration, neuroinflammation, and secondary mechanisms altering vagal–sympathetic balance [38,39,40].

Epidemiologic data indicate that AD is frequent across amyloid subtypes, though with different phenotypic patterns and timing. Screening studies reveal a prevalence of CA as high as 13–15% among older patients with HFpEF [41]. Registry data demonstrate that early autonomic symptoms—orthostatic hypotension (OH), gastrointestinal (GI) dysmotility, erectile dysfunction (ED)—are reported in up to 70% of Val30Met and Glu89Gln carriers, supporting the concept of autonomic neuropathy (AN) as a prodromic phenotype [1,29]. The impact on quality of life (QoL) is profound: validated patient-reported measures such as the ATTR-QoL questionnaire confirm that dysautonomia significantly impairs daily functioning and psychological well-being, independently of cardiac stage [42].

Genetic determinants influence the distribution and severity of autonomic dysfunction. Mutations associated with neuropathic ATTRv variants (Val30Met, Thr60Ala, Ser77Tyr) typically present with early and symmetric, length-dependent diffuse AuF with severe OH and GI dysmotility, reflecting both small-fiber and vagal degeneration [31,40]. In contrast, variants such as Val122Ile or ATTRwt more often manifest with predominant cardiac involvement and with relatively preserved extracardiac autonomic function, or with late-onset and mild dysautonomia [1,30,43]. Conversely, AL exhibits heterogeneous autonomic involvement, determined both by amyloid burden and by the intrinsic neurotoxicity of circulating light chains [8,44]. In rare systemic forms such as AA amyloidosis, AN is described but usually less pronounced [45].

Although the exact mechanisms underlying the greater burden of AD in ATTRv compared with ATTRwt remain incompletely defined, several observations offer plausible explanations. Neuropathic ATTRv variants such as Val30Met, Thr60Ala, and Ser77Tyr show early and preferential involvement of small autonomic fibers and autonomic ganglia, with evidence of amyloid deposition, axonal loss, and Schwann cell injury [38,39,40]. Moreover, pre-fibrillar TTR aggregates appear to exert direct neurotoxic effects on peripheral autonomic structures. By contrast, ATTRwt is characterized by a predominantly cardiac phenotype, with limited extracardiac autonomic involvement and later-onset dysautonomia [1,43].

Thus, while a definitive model is still lacking, the combination of neurotropic variant-specific biology and diffuse peripheral nerve infiltration likely contributes to the more severe autonomic impairment observed in hereditary forms.

Comparative evidence suggests that AD in amyloidosis often reflects combined cardiovagal and adrenergic failure, whereas infiltrative or metabolic cardiomyopathies (e.g., Fabry disease, sarcoidosis, hemochromatosis) exhibit distinct autonomic profiles [39,40,41,42,43,46,47,48,49,50].

Histopathological and imaging evidence demonstrate amyloid deposition along the entire autonomic pathway— affecting the VN, sympathetic ganglia, and postganglionic fibers—with intrinsic enteric plexus degeneration [38,40]. Amyloid infiltration within intramural ganglia and the ICNS disrupts local reflex arcs that normally modulate sinus node activity and atrioventricular conduction, leading to progressive vagal denervation [51].

Beyond structural deposition, experimental data and early stage data support a neurodegenerative component: human biopsies demonstrated axonal loss and toxic prefibrillar TTR aggregates activating RAGE-mediated oxidative and inflammatory pathways [36,37].

The concept of amyloidosis as a neuroinflammatory disease has gained support. Emerging evidence supports the view that amyloidosis entails a significant neuroinflammatory component that extends beyond peripheral neuropathy to involve central autonomic regulatory circuits. Experimental and translational studies [11,38,39,52] support a neuroinflammatory component involving both circuits. Microglial activation within key regulatory nuclei contributes to cytokine release, oxidative stress, and progressive autonomic decline, while peripheral nerves exhibit analogous inflammatory injury. Moreover, recent observations highlight that the heart–brain axis may represent a bidirectional interface through which systemic amyloid burden and central neuroinflammation interact, potentially amplifying AD and cardiovascular dysfunction [11].

Disruption of these mechanisms may amplify neuroinflammation, reinforcing a cycle of autonomic impairment.

Autonomic denervation has direct implications for neurocardiac control and arrhythmogenesis. Loss of vagal input destabilizes sinus node automaticity and predisposes to atrial fibrillation (AF), whereas sympathetic imbalance promotes ventricular ectopy and sudden cardiac death [33,53]. Clinical studies describe a high prevalence of bradyarrhythmias, chronotropic incompetence, and atrioventricular block in both ATTR and AL amyloidosis, often requiring pacemaker implantation [32,54]. AF affects nearly half of patients with ATTR-CA and correlates with greater amyloid burden and diminished HRV indices [53,55]. Together with OH and impaired baroreflex buffering, these mechanisms contribute to exercise intolerance, syncope, and deterioration in QoL [29,32].

Prognostically, AuF represents a major determinant of outcome. Reduced cardiac vagal tone and generalized AuF predict higher mortality across amyloid subtypes [8,37]. After liver transplantation in ATTRv, persistence of cardiac dysautonomia identifies patients at the highest risk of progression [37].

Similarly, in AL amyloidosis, neurofilament light chain (NfL) levels correlate with the severity of neuropathy, autonomic decline, and poor survival, serving as a potential biomarker [35,44]. Beyond prognostic value, AD may directly contribute to adverse events: cardiovagal impairment and conduction disease increase the risk of syncope-related falls, while OH directly predisposes to presyncope, syncope, and postural instability [31,56,57].

These findings suggest that AuF in amyloidosis influences prognosis not only as a risk marker but also through specific pathophysiological mechanisms that increase susceptibility to cardiovascular and traumatic complications.

## 4. Clinical Manifestations of Autonomic Involvement

AD in CA encompasses a broad spectrum of clinical manifestations that frequently precede overt cardiomyopathy and represent an early warning sign of systemic neural involvement. The pattern and severity of manifestations differ among amyloid subtypes and depend on the balance between vagal and sympathetic fiber degeneration [9,31,58].

**Figure 1:**
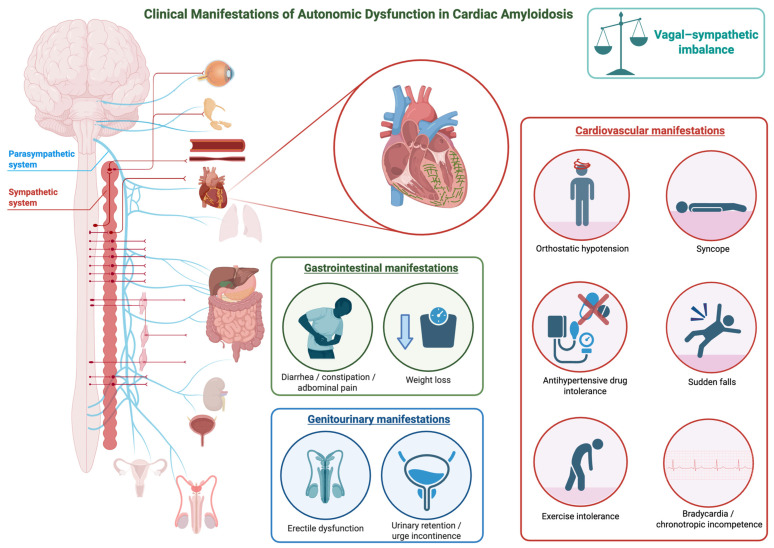
Clinical Manifestations of Autonomic Dysfunction in Cardiac Amyloidosis. The figure illustrates the main clinical manifestations of autonomic dysfunction in cardiac amyloidosis. This figure was created with BioRender.com.

The main clinical manifestations of AD in CA are illustrated in Figure 1.

Early warning manifestations frequently dominate the clinical onset of ATTRv. OH is among the most common and disabling symptoms, reported in up to two-thirds of affected individuals [31]. Patients experience dizziness, visual blurring, or syncope upon standing. These episodes result from the inability to maintain adequate blood pressure and heart rate when moving to an upright position, reflecting combined adrenergic and vagal failure [5,57]. Recurrent presyncope or syncope—sometimes unpredictable—interferes severely with daily activities and is a hallmark of advanced autonomic impairment [4,34,36].

Exercise intolerance and fatigue are also frequent early complaints. Patients describe reduced capacity for physical effort, heat intolerance, and a sense of exhaustion even after minimal exertion. These symptoms stem from inadequate cardiovascular adaptation and diminished parasympathetic withdrawal during exercise. Antihypertensive drug intolerance also occurs. Episodes of bradycardia or chronotropic incompetence may accompany this reduced tolerance, emphasizing the link between loss of vagal tone and exertional limitation [9,58].

Syncope, which may occur spontaneously or in association with exertion, coughing, or meals, often anticipates more severe cardiac conduction disturbances [4,32]. It is particularly frequent in ATTRv and AL and may recur despite otherwise preserved systolic function. The coexistence of syncope and orthostatic intolerance should prompt suspicion of diffuse autonomic neuropathy [6,57].

Visceral manifestations are a major component of AD in CA and tend to be more pronounced in patients with ATTRv. In these forms, GI, genitourinary, and sudomotor symptoms—including diarrhea, constipation, nausea, early satiety, and erectile or urinary dysfunction—frequently coexist and reflect diffuse involvement of autonomic pathways rather than isolated organ impairment. In contrast, such manifestations are usually mild or absent in non-hereditary subtypes [5,6,57].

GI disturbances represent a major source of morbidity. Early satiety, postprandial fullness, nausea, vomiting, and abdominal bloating are commonly reported [5,31]. Alternating diarrhea and constipation occur in advanced stages and reflect disordered intestinal motility. Diarrhea may be severe and persistent, leading to dehydration and weight loss, while constipation contributes to abdominal discomfort and bloating. These symptoms often fluctuate over time, a pattern consistently described in multiple clinical studies and attributed to the dynamic imbalance of autonomic control along the GI tract [5,6,57]. Genitourinary dysfunction is frequent and significantly impairs quality of life. ED is often one of the earliest manifestations of parasympathetic failure, while urinary retention or urge incontinence appear as the disease progresses. These symptoms highlight the combined loss of sympathetic and parasympathetic modulation of the pelvic organs [5,31,57].

Sudomotor symptoms provide additional evidence of small-fiber and AuF. Patients frequently report dry skin, reduced or absent sweating, and heat intolerance, which evolve from distal to proximal regions. These changes are accompanied by sensations of dryness in the eyes and mouth, impaired thermoregulation, and chronic fatigue [57].

The “vagal link” concept provides an interpretive framework for understanding autonomic manifestations in CA. Degeneration of the VN, which regulates heart rate, GI motility, and visceral homeostasis, gives rise to a characteristic constellation of symptoms—bradycardia, exercise intolerance, early satiety, and alternating diarrhea and constipation—that define a “vagal phenotype” of AuF, particularly evident in early-onset ATTRv [9,31].

Across amyloid subtypes, ATTRv exhibits early and diffuse AN with multisystemic symptoms such as OH, GI dysmotility, ED, and anhidrosis [5,31]. AL displays a variable pattern, ranging from mild orthostatic intolerance to severe cardiovascular and GI dysfunction, depending on disease burden [4,9]. In contrast, ATTRwt presents cardiac-restricted dysautonomia, with late-onset symptoms—syncope, exertional fatigue, and orthostatic intolerance—reflecting localized cardiovagal and sympathetic impairment [58].

## 5. Diagnostic Tools for Autonomic Dysfunction

**Figure 2:**
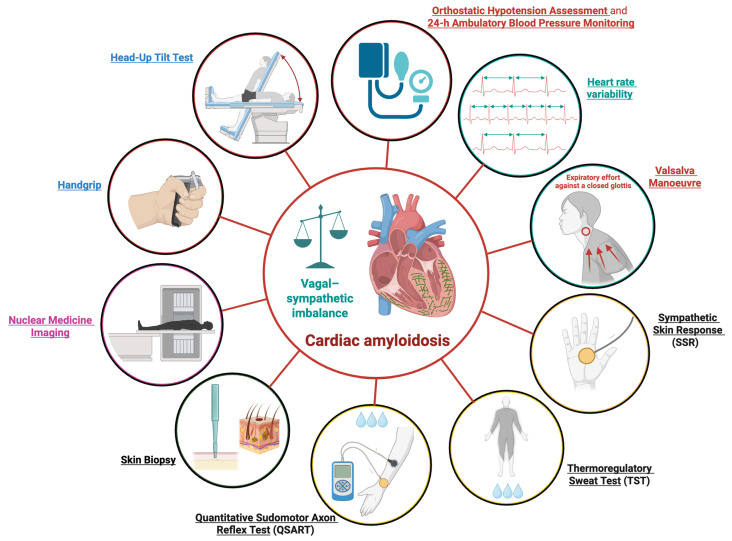
Diagnostic Assessments for Autonomic Dysfunction in Cardiac Amyloidosis. Schematic overview of the principal modalities used to evaluate autonomic dysfunction in cardiac amyloidosis; 24-h, 24 h. This figure was created with BioRender.com.

Several tools and diagnostic modalities are currently available to assess autonomic dysfunction. These belong primarily to neurological and cardiological evaluations, although nuclear medicine imaging can also be employed. Such approaches are particularly valuable for assessing this condition in patients with CA. The principal modalities used to evaluate AD in CA are schematically illustrated in Figure 2.

The following sections describe these diagnostic methods, grouped according to their respective clinical domains.

### 5.1. Neurological Evaluation

Autonomic neurophysiological tests play a crucial role in the objective assessment of ANS function. Autonomic tests focus on assessing small-caliber myelinated fibers (Aδ) and unmyelinated fibers (C) responsible for autonomic functions [59]. According to the American Autonomic Society and the International Federation of Clinical Neurophysiology, standardized autonomic tests study three functional domains: cardiovagal (parasympathetic) function, adrenergic (sympathetic) function, and sudomotor (sympathetic cholinergic) function [59]. The integration of tests that assess these domains contributes to the Composite Autonomic Severity Score (CASS), a reliable quantitative index of autonomic impairment [60]. In addition to the functional assessment of the ANS using neurophysiological tests, it is also possible to perform a structural evaluation of autonomic innervation through histological examination of the skin [61].

#### 5.1.1. Heart Rate Variability

HRV analysis is the most widely validated non-invasive method for assessing parasympathetic (cardiovagal) function [62]. It measures the physiological fluctuation in the time intervals between consecutive heartbeats (R–R intervals), reflecting the dynamic balance between sympathetic and parasympathetic cardiac control. HRV evaluations can be performed in both the time and frequency domains [63].

In clinical settings, the Heart Rate Response to Deep Breathing (HRDB) test is the most standardized and widely used method for assessing cardiovagal function. During HRDB testing, the patient breathes while continuous ECG monitoring records beat-to-beat heart rate changes. The standard analysis involves calculating the E–I difference and the E–I ratio. Test results are compared to age-adjusted normative values; a reduced E–I difference or E–I ratio indicates parasympathetic dysfunction, which is typical of patients with systemic amyloidosis with ANS involvement [27,62,64]. HRV is a prevalent metric employed for the evaluation of autonomic function [65] and has been established as a prognostic indicator in CA [9,66,67]. AD is commonly documented in both TTR and AL amyloidosis. Notably, a reduction in the SDRR to <50 ms serves as a robust predictor of one-year mortality in patients afflicted with amyloidosis [66]. Despite standardized measurement conditions, the clinical utility of short-term HRV is constrained by its inherent variability. Consequently, the coefficient of variation (CV) of the R-R interval (CVR-R) has been proposed as a more stable alternative metric for assessment [68]. Several studies have analyzed this parameter in patients with CA. Koike et al. [67]. evaluated cardiac autonomic functions in 5 non-Val30Met patients [67]. The CVR-R ratio (alongside the Valsalva ratio) was found to be abnormal in all patients examined. These concurrent findings definitively indicate the presence of widespread cardiac parasympathetic dysfunction within the study population [59]. In a subsequent study by Nagayoshi et al. [69] conducted on 50 patients with HF, including 10 patients with ATTRwt, it was demonstrated that the lowest CVR-R levels were specifically observed in the patients afflicted with amyloidosis [69]. HRV is therefore a non-invasive and safe examination, which is relatively accessible and cost-effective. However, its utility is constrained by high inherent variability and a lack of specificity within certain frequency domains that simultaneously reflect both sympathetic and vagal modulation.

#### 5.1.2. Valsalva Maneuver

The Valsalva Maneuver is a non-invasive test of both cardiovagal and adrenergic function, providing valuable insight into baroreflex integrity. The patient performs a forced expiratory effort (approximately 40 mmHg for 15–20 s) against a closed glottis, while continuous beat-to-beat heart rate and blood pressure are recorded [59]. The characteristic hemodynamic response consists of four phases. The Valsalva Ratio, the maximal heart rate during the maneuver divided by the minimal heart rate after the release, is a quantitative measure of cardiovagal function. A reduced or absent overshoot in Phase IV indicates sympathetic adrenergic failure, while a diminished Valsalva ratio reflects parasympathetic impairment [59]. In systemic amyloidosis, the Valsalva Maneuver frequently shows a reduced increase in heart rate in phase II and an absent or delayed blood pressure recovery in phase IV, indicative of combined parasympathetic and sympathetic failure [8,27]. Crucially, the resultant change in heart rate elicited by this maneuver is a widely accepted, indirect, sensitive, specific, and reproducible measure of cardiac parasympathetic autonomic function [70]. A key advantage of the maneuver is its capacity for a dual-function assessment of both parasympathetic and sympathetic cardiac control, providing a highly sensitive and specific diagnostic tool that is both safe and non-invasive, particularly for identifying the combined autonomic failure frequently observed in conditions like CA. However, its clinical accuracy is inherently limited by patient cooperation in maintaining the necessary expiratory effort and the absolute requirement for specialized, continuous beat-to-beat monitoring. Furthermore, the resultant increase in intrathoracic and intraocular pressure necessitates that the maneuver is contraindicated or requires stringent caution in patients with certain pre-existing conditions.

#### 5.1.3. Sympathetic Skin Response

The Sympathetic Skin Response (SSR) is a neurophysiological test used to evaluate the sympathetic cholinergic sudomotor pathway [71]. When an unexpected stimulus is presented (electrical, auditory, emotional, or deep inspiration), a change in skin potential due to the activation of sweat glands innervated by cholinergic sympathetic fibers can be recorded, usually from palmar and plantar sites [71]. Amplitude and latency of the response can be measured; however, given the considerable inter-individual and intra-individual variability of the response, in clinical practice, the evaluation is limited to determining whether the response is present or absent. In amyloid-related autonomic neuropathy, SSR is often absent or exhibits markedly reduced amplitude, reflecting loss of sympathetic postganglionic integrity [72].

#### 5.1.4. Thermoregulatory Sweat Test

The Thermoregulatory Sweat Test (TST) provides an assessment of central and peripheral sympathetic sudomotor function by evaluating the body’s ability to produce sweat in response to a standardized thermal stimulus [73,74]. TST is rarely used in routine clinical practice due to its technical complexity and specialized equipment required.

#### 5.1.5. Quantitative Sudomotor Axon Reflex Test

The Quantitative Sudomotor Axon Reflex Test (QSART) is considered the gold standard for evaluating postganglionic sympathetic cholinergic function. It measures localized sweat output in response to a cholinergic agent [75]. The latency of the sweat onset, the maximal sweat volume produced, and the area under the curve of sweat production over time are then measured by hygrometers placed at standardized sites. Absent or reduced sweat production indicates postganglionic sympathetic dysfunction [74]. In systemic amyloidosis, QSART commonly shows a length-dependent reduction or absence of sweat production. Given the quantitative nature of the test, QSART offers a valuable tool for longitudinal monitoring and for evaluating the response to treatment in patients with amyloidosis [8,74]. The clinical utility of the test is, however, limited by its technical nature, necessitating specialized equipment and highly trained personnel. Furthermore, it entails a degree of mild invasiveness due to the requirement for iontophoresis. Crucially, while serving as the benchmark for sudomotor assessment, QSART only reflects this single autonomic function, thereby precluding the concurrent evaluation of other essential branches, such as cardiovagal or adrenergic control.

#### 5.1.6. Skin Biopsy

Skin biopsy is a minimally invasive technique to assess the innervation of the skin by small somatic and autonomic fibers. Small punch biopsies are typically obtained from distal and proximal sites (e.g., distal leg and thigh). Biopsies are then processed for immunohistochemical staining using markers that target nerve fibers and other components of the skin [61]. This technique allows the visualization and quantification of intraepidermal nerve fibers (IENFs) as well as sudomotor nerves, pilomotor nerves, and vasomotor nerves. The evaluation of sudomotor innervation density provides valuable information about postganglionic sympathetic function, and its reduction, together with a reduction in the density of vasomotor and pilomotor innervation, is useful for the diagnosis of autonomic neuropathy [61,76]. Reduced sudomotor innervation has also been documented in patients with ATTRv, in whom it correlates with the presence of autonomic symptoms and alterations in autonomic testing [77].

### 5.2. Cardiological Evaluation

The evaluation of cardiovascular autonomic function constitutes the fundamental and most critical component of the clinical investigation into overall autonomic function. From a cardiovascular perspective, the evaluation of ANS function in patients with amyloidosis commonly employs HRV analysis and the Valsalva Maneuver, both of which have been previously described.

#### 5.2.1. Orthostatic Hypotension Assessment and Head-Up Tilt Test

The evaluation of AD via OH is predicated on the substantial drop in blood pressure observed upon a patient assuming an upright posture. The established diagnostic criteria for clinically significant OH mandate a sustained decrease of >20 mmHg in systolic blood pressure (SBP) or >10 mmHg in diastolic blood pressure within 3 min of standing or the head-up tilt test (HUTT) [56,78,79]. The standardized protocol requires the patient to remain supine or seated for a minimum of five minutes to attain hemodynamic stability. Following this baseline period, the patient transitions rapidly to standing or is passively tilted upright for a duration of one to three minutes, during which systolic blood pressure, diastolic blood pressure, and heart rate are concurrently recorded to quantify the orthostatic response [80].

In 2023, Guaraldi and colleagues executed an investigation involving a cohort of patients diagnosed with amyloidosis, who were subsequently stratified based on the presence or absence of polyneuropathy, alongside a comparator group of healthy controls [27]. The methodology of this study encompassed multiple diagnostic procedures, notably the head-up tilt test to evaluate OH. Of the 37 subjects with ATTRv, four (11%) showed a BP drop that meets the criteria for OH [27]. The authors highlighted, however, that AD was quite apparent even in subjects who did not exhibit OH, which underscores the superior sensitivity of a comprehensive panel of standardized cardiovascular autonomic tests, particularly those incorporating cardiovagal assessment, when compared solely to blood pressure measurements taken during a HUTT or standing [27]. Referring again to the previously cited study by Koike and colleagues, the authors found that the total peripheral resistance at 60° head-up tilt failed to increase relative to the supine baseline in all patients evaluated [67], when the sole clinical parameter of OH was not recorded in all the patients. This lack of a compensatory vasoconstrictor response, which is physiologically requisite for the maintenance of blood pressure upon orthostasis, conclusively demonstrated a pervasive failure of peripheral vasomotor autonomic function across the entire cohort, regardless of whether the clinical criterion for OH was met [67]. Similar findings were reported in a previous study by Koike and colleagues on eight patients with ATTRv Val30Met amyloidosis [81]. Consistent with the previous investigation, not all patients developed OH based solely on clinical criteria. However, a significant observation was the failure to achieve an increase in total peripheral resistance from baseline during the 60° head-up tilt test in five of the six patients who underwent this specific hemodynamic measurement [81]. This observation definitively established a widespread peripheral vasomotor AD within the cohort and highlighted the potential role of the peripheral vascular resistance changes during the tilt test [81].

#### 5.2.2. Twenty-Four-Hour Ambulatory Blood Pressure Monitoring

Blood pressure (BP) is regulated by the balance of ANS activity throughout the day and night, influencing both heart rate (HR) and BP levels. Normally, BP follows a circadian rhythm, exhibiting higher values during periods of wakefulness and a characteristic nocturnal descent [82]. A blunted or reversed nocturnal BP pattern—where the expected nighttime decrease is diminished or absent—signifies an exacerbated sympathetic nervous system tone. This dysregulation is frequently observed in patients with dysautonomia and has been robustly linked to increased mortality risk [83]. Ambulatory Blood Pressure Monitoring (ABPM) is a valuable diagnostic tool that extends beyond conventional BP measurements. It is particularly effective for identifying clinically relevant conditions such as nocturnal hypertension, which is a significant predictor of adverse cardiovascular events [84]. In patients diagnosed with amyloidosis, the observation of supine hypertension coupled with an abnormal circadian blood pressure pattern on ABPM strongly suggests underlying dysautonomia [57].

#### 5.2.3. Isometric Exercise

Isometric exercise precipitates a reflex-mediated elevation in mean arterial pressure and cardiac frequency [85]. The hemodynamic alterations provoked by a sustained handgrip maneuver have been clinically adopted as an index for assessing sympathetic nervous system function [86,87]. The standard isometric handgrip test is used to elicit a sympathetic response, but the results are prone to marked variability. This inconsistency is partially due to the difficulty in precisely standardizing the relative muscular effort. Furthermore, evidence indicates that exercise training can diminish muscle afferent signaling, consequently attenuating the associated sympathetic outflow and the expected pressor response normally observed during static exercise [87]. In a 2022 study by Di Stefano et al., the investigators reported a significant reduction in handgrip strength (HGS) in both the right and left hands of patients with ATTRv amyloidosis when compared to healthy control subjects [88]. However, a subsequent investigation by the same group, evaluating HGS in ATTRv patients receiving patisiran therapy, revealed no significant change in HGS after nine months of treatment [89]. This finding contrasted with other measures, such as bioelectrical impedance analysis, which showed a measurable change over the same period. Despite its utility for research, the test’s application in clinical diagnosis is constrained by its poor sensitivity and specificity [70].

### 5.3. Nuclear Medicine Imaging

#### 5.3.1. Scintigraphy and SPECT Imaging

**Figure 3:**
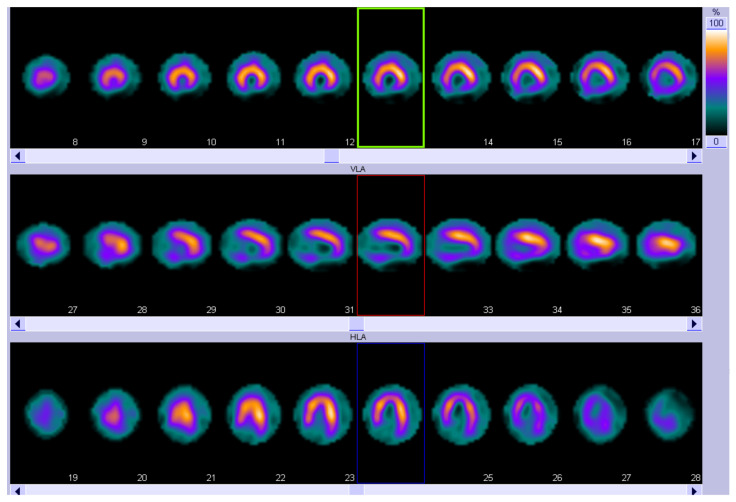
^123^I-MIBG SPECT showing an extensive area of reduced innervation in the inferior apex and inferior wall of the left ventricle. Short-axis view (top row), vertical long-axis view (middle row), and horizontal long-axis view (bottom row). MIBG, Meta-iodobenzylguanidine; SPECT, Single Photon Emission Computed Tomography.

It is well established that ^99m^technetium-pyrophosphate (^99m^Tc-PYP), 3,3-diphosphono-1,2-propanodicarboxylic acid (DPD), and hydroxymethylene diphosphonate (HMDP) bone-tracer scintigraphy with Single Photon Emission Computed Tomography (SPECT) represent a cornerstone in the diagnosis of ATTR-related CA [90,91,92,93]. More recently, in addition to standard neurophysiological and cardiological assessments, nuclear imaging techniques have been used to evaluate cardiac dysautonomia in patients with amyloidosis. Meta-iodobenzylguanidine (MIBG) is a noradrenaline analog labeled with ^123^I to allow for imaging, reflecting the density, integrity, and function of postganglionic noradrenergic neurons [94]. SPECT images are usually acquired to evaluate the regional cardiac sympathetic denervation (Figure 3).

**Figure 4:**
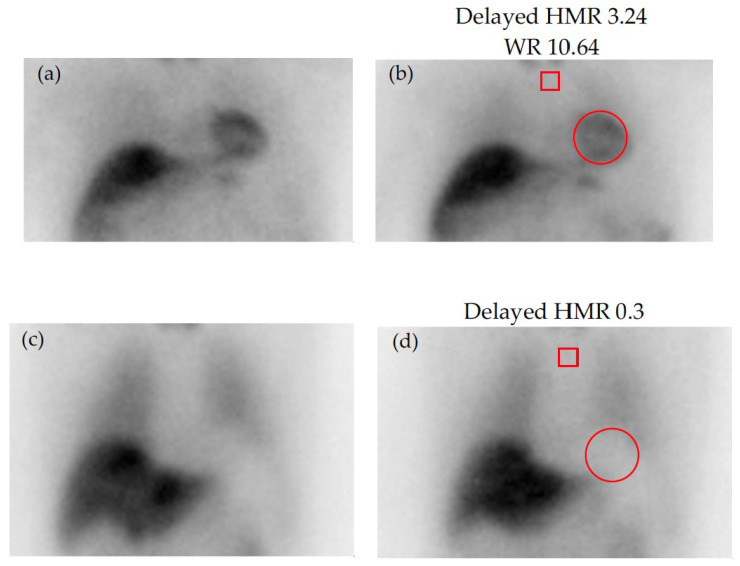
Early (**left**) and delayed (**right**) static images with ^123^I-MIBG: (**a**,**b**) normal scan; (**c**,**d**) absent cardiac tracer uptake. Regions of interest (ROI) are placed on the upper mediastinum (red square) and the heart (red circle) for quantification of tracer uptake. HMR = heart-to-mediastinum ratio (mean counts per pixel in the cardiac ROI by the mean counts per pixel in the mediastinal ROI); MIBG, Meta-iodobenzylguanidine; WR = washout rate (ratio of cardiac uptake between early and delayed scans).

For quantification, early (10 min post-injection) and delayed (3–4 h post-injection) acquisitions are performed, and the MIBG washout rate (WR) is calculated as the ratio of cardiac uptake between early and delayed scans. The WR is thought to reflect the turnover of catecholamines and sympathetic tone. Tracer accumulation in the left ventricle compared to that for the upper mediastinum from anterior planar images (heart-to-mediastinum ratio, HMR) is also quantified and provides information regarding the uptake, storage, and release of MIBG at nerve terminals [95] (Figure 4).

Patients with CA tend to show a reduced HMR and a higher WR, compared to healthy subjects, indicating an impaired 123I-MIBG uptake and thereby an impairment of the cardiac sympathetic function due to amyloid infiltration. In a study conducted by Coutinho et al. [96] involving 143 individuals carrying the Val30Met transthyretin mutation, cardiac sympathetic denervation assessed by MIBG imaging emerged as a valuable prognostic marker in transthyretin familial amyloid polyneuropathy [96]. Patients exhibiting cardiac sympathetic denervation, defined by a late HMR below 1.60, were identified as being at the highest risk for adverse clinical outcomes [96].

In patients diagnosed with ATTRv amyloidosis, the detection of abnormal myocardial MIBG uptake and accelerated washout is directly correlated with cardiac sympathetic denervation, a pathological outcome resulting from the ongoing degeneration of autonomic nerve fibers [97]. In patients with familial amyloid polyneuropathy, a high prevalence of myocardial adrenergic denervation has been documented despite preserved myocardial viability. This alteration can be detected at an early stage of CA, preceding the onset of clinically evident cardiac involvement, ventricular wall thickening, or significant left ventricular systolic and diastolic dysfunction [97,98] Piekarski et al. [99] have highlighted a key temporal relationship in the progression of amyloidosis: cardiac sympathetic denervation, as reflected by diminished MIBG uptake, frequently becomes evident before the measurable accumulation of amyloid protein, which is typically quantified using DPD-scintigraphy. More recently, the same observation has been reported by Jonker et al. in 39 patients (13 carriers and 26 ATTR patients) [100].

These results confirm that both cardiac bone-seeking tracer accumulation and decreased late HMR on 123I-MIBG scintigraphy reflect cardiac involvement of amyloidosis. Moreover, these findings also suggest a significant and promising role for MIBG scintigraphy in the early identification of cardiac involvement in asymptomatic or early-stage carriers of the TTR gene mutation [91]. According to Feo et al. [101] in a study investigating cardiac sympathetic denervation in patients with ATTRv amyloidosis using I-123 MIBG scintigraphy, this imaging technique proved to be an effective tool for assessing cardiac denervation. Given the potential of cardiac denervation to increase the risk of malignant arrhythmias and sudden cardiac death—particularly in patients with ATTRv amyloidosis—123I-MIBG scintigraphy may also represent a valuable method for risk stratification [93]. A prospective study conducted by Nicol et al. [102] evaluated 52 patients with biopsy-proven AL amyloidosis to assess cardiac denervation using 123I-MIBG scintigraphy. The analysis demonstrated that cardiac denervation is a common and often severe manifestation in AL amyloidosis. Indeed, cardiac denervation as assessed by 123I-MIBG was a frequent finding, severe in most patients. From analyses of receiver-operating characteristic curves, a late H/M ratio ≤ 1.44 was significantly associated with a poorer overall prognosis [102].

#### 5.3.2. Positron Emission Tomography (PET)

PET leveraging tracers that target sympathetic innervation, such as ^11^C-hydroxyephedrine (^11^C-mHED) or ^11^C-epinephrine, represents a more advanced imaging methodology compared to ^123^I-MIBG scintigraphy. This is due to the inherent superiority of PET in several key technical aspects, including enhanced sensitivity and spatial resolution, alongside the capability for absolute quantitative analysis enabled by the routine integration of attenuation and scatter correction techniques [103]. ^11^C-mHED is a compound derived from metaraminol, a false norepinephrine analogue [104,105]. In the case of sympathetic de-innervation, there is an increase in spillover of ^11^C-mHED from the nerve terminal, leading to a decreased reuptake [106]. To date, there is a paucity of research directly comparing ^11^C-mHED and ^123^I-MIBG. A significant limitation of ^11^C-mHED tracer is the logistical requirement of access to a local cyclotron [107], due to the very short half-life of the PET isotope ^11^C, which drastically reduces the number of centers able to perform this examination and therefore its large-scale clinical applicability.

In conclusion, nuclear techniques provide multimodal and effective tools for the noninvasive diagnosis of ATTR-related CA with bone-avid tracers and the assessment of cardiac denervation in TTR and AL amyloidosis with ^123^I-MIBG. Moreover, MIBG SPECT proved to be an independent prognostic marker of poor outcome in ATTRh-CA [108]. However, available studies using MIBG imaging in patients with CA include relatively small patient cohorts. Identify the presence of denervation even before bone-avid scintigraphy. Future investigations will be important to further elucidate the diagnostic and prognostic potential of these modalities and to advance toward a more specific and individualized diagnostic and clinical approach for affected patients.

## 6. Prognostic and Therapeutic Implications

The manifestation of cardiovascular AD in the context of CA encompasses a constellation of symptoms, including neurogenic OH [109], syncope, and postural dizziness [110]. This condition’s characteristic autonomic denervation of the myocardium further predisposes patients to the development of significant cardiac arrhythmias and intraventricular conduction defects [110]. The presence of generalized AuF demonstrated by autonomic testing is an independent adverse prognostic factor for survival in patients with AL amyloidosis [66].

Evidence also indicates that a reduction in HRV is an early and reproducible marker of cardiovagal failure in amyloidosis, and it is correlated with poorer clinical outcomes, predominantly observed in AL amyloidosis [10]. Decreased heart-to-mediastinum ratios on late MIBG images and increased wash-out rates indicate cardiac sympathetic denervation and are associated with poor prognosis [103].

Management approaches for this pathology follow a structured progression. Initial efforts focus on non-therapeutic measures (lifestyle modifications). The subsequent phase involves the administration of pharmacological agents targeting specific autonomic manifestations, such as those used to treat OH and promote vagal enhancement. The most advanced interventions constitute disease-modifying drugs that directly interrupt the underlying pathology by targeting amyloid deposition while simultaneously providing symptomatic relief [111,112].

### 6.1. Nonpharmacological Measures

Non-pharmacological management constitutes a foundational strategy for symptom mitigation in autonomic dysfunction. These interventions primarily center on the modification of dietary intake: specifically, minimizing the ingestion of diuretic and volume-depleting substances, such as caffeine and ethanol, while simultaneously enhancing fluid and sodium consumption to promote effective intravascular volume expansion in patients afflicted by OH [113,114]. Palma et al. [56] recommend a daily fluid intake of approximately 2.0–2.5 L, with a corresponding recommendation for patients to augment their dietary sodium by integrating one to two teaspoons of supplemental salt into their regimen [48]. Importantly, such measures of volume expansion are contraindicated in patients presenting with clinical signs of congestive HF, in whom fluid and sodium restriction remains essential [115,116]. Therefore, a crucial aspect in this patient population is achieving a delicate and often unstable balance between the need for volume expansion and the requirement to maintain fluid restriction. Furthermore, the use of external compression garments has demonstrated clinical benefit in managing OH [56,113,114,117,118]. In particular, high-waist compression stockings providing at least 15–20 mmHg of pressure can be employed to support blood pressure by enhancing venous return to the central circulation [119].

### 6.2. Medication for Neurogenic Orthostatic Hypotension

Despite the rigorous and appropriate implementation of non-pharmacological strategies, a significant proportion of patients ultimately requires pharmacological intervention to mitigate symptomatic OH. Notably, the early development of AD and OH represents an independent predictive factor for reduced survival in ATTRv [120].

The established therapeutic paradigm integrates two distinct, yet complementary, mechanisms of action: intravascular volume expansion and the elevation of peripheral vascular resistance. The foundational component of the volume expansion strategy is typically predicated upon the administration of fludrocortisone, a synthetic mineralocorticoid agent that increases intravascular volume and consequently elevates systemic blood pressure [121]. While fludrocortisone remains extensively employed in patients with OH [122], its usage is associated with an increased risk of fluid retention exacerbation in those afflicted by CA [113,114]. Furthermore, its administration may correlate with a heightened risk of all-cause hospitalization when compared to the use of midodrine [123].

The second pharmacological strategy focuses on the elevation of peripheral vascular resistance; an effect primarily achieved through the use of midodrine in patients with OH [122]. Midodrine functions as an oral α1-adrenoreceptor agonist, thereby inducing systemic vasoconstriction and increasing blood pressure [124]. Vasoconstrictive agents, such as midodrine, prove beneficial in patients with CA by helping to maintain adequate blood pressure, enhancing diuresis, and consequently facilitating the maintenance of adequate diuretic dosages [3,56]. In specific clinical scenarios, notably in cases of ATTRv, the administration of midodrine may be beneficial, particularly when high or escalating doses of diuretics are necessary [125]. Nevertheless, caution is advised when utilizing midodrine in patients with HF. Retrospective investigations have indicated that the use of midodrine in patients presenting with a left ventricular ejection fraction (LVEF) of less than 35% is correlated with an unfavorable prognosis [126]. Notably, the supine hypertension commonly associated with midodrine administration is infrequent in CA patients, permitting the use of higher dosages in this patient population [127].

Droxidopa, an oral synthetic amino acid, functions as a prodrug that is enzymatically converted to norepinephrine [128]. In 2014, the US Food & Drug Administration (FDA) approved droxidopa for the management of symptomatic stemming from pure AuF and non-diabetic autonomic neuropathy, a category that encompasses ATTRv and other etiologies of autonomic neuropathy [129]. Although the extant corpus of research on droxidopa utilization in individuals with amyloidosis is limited, a small-scale retrospective observational study evaluating its effects in critically ill hospitalized patients demonstrated improvements in those with amyloidosis [130]. Furthermore, multiple case reports have demonstrated the medication′s utility in mitigating OH in patients presenting with AL amyloidosis [129,131]. Droxidopa appears to confer a lower risk of supine hypertension compared to midodrine, with nausea and headache constituting the most frequently reported adverse effects [132,133].

### 6.3. Enhancing Autonomic Systems

Pyridostigmine has been demonstrated to selectively elevate upright blood pressure in patients diagnosed with OH [134]. The hypothesized mechanism of action involves the enhancement of cholinergic neurotransmission at the autonomic ganglia. This effect is mediated by the inhibition of cholinesterase, which consequently potentiates local acetylcholine concentrations [135]. The 2023 ACC consensus document supports the integration of pyridostigmine into the therapeutic armamentarium for OH, particularly noting the favorable absence of supine hypotension as an adverse effect [136]. While this agent is frequently cited in management guidelines for patients afflicted with either TTR or AL amyloidosis [137,138], its efficacy and safety have not been specifically investigated in individuals with CA.

### 6.4. Disease-Modifying Therapies and the Effects on Autonomic Systems

Diflunisal, a nonsteroidal anti-inflammatory drug (NSAID), operates through the stabilization of circulating transthyretin tetramers. This specific mechanism precludes the dissociation into constituent monomers, a critical prerequisite for subsequent amyloidogenesis [139]. Its clinical efficacy was substantiated in a 2–year double–blind, placebo–controlled trial, which demonstrated that diflunisal attenuated the rate of neurological impairment progression and preserved patient QoL in individuals with ATTRv [140].

Tafamidis, a TTR stabilizer, has been documented to attenuate the progression of variant transthyretin amyloid polyneuropathy [141,142]. Nevertheless, its therapeutic efficacy in managing dysautonomia remains inconsistent across both published clinical trials and real-world observational data. Despite this variability, the available evidence indicates the agent is well-tolerated and may circumstantially slow the advancement of AN in select patient subsets [143]. This potential benefit complements its established effect on slowing the deterioration of sensory-motor peripheral neuropathy [144]. Consequently, this therapeutic agent may confer an advantage to individuals with CA who develop subsequent neurological manifestations, but who did not initially present with an exclusively polyneuropathic phenotype. Acoramidis, another TTR stabilizer, has also been shown to be effective in reducing cardiovascular events and mortality in patients with transthyretin amyloid cardiomyopathy. To our knowledge, patient recruitment is currently underway for a randomized clinical trial (NCT06563895) aimed at evaluating whether the drug is capable of preventing or delaying the development of CA or polyneuropathy [145].

TTR gene silencers function by suppressing expression through post-transcriptional inhibition, thereby fundamentally curtailing the synthesis of the protein. This therapeutic modality encompasses small nucleic acid-based molecules, specifically small interfering RNAs (siRNA), such as patisiran and vutrisiran [146,147], and antisense oligonucleotides (ASOs), exemplified by inotersen and eplontersen [148,149,150]. Collectively, these agents mitigate transtiretin production, effectively limiting the precursor substrate required for amyloid fibril deposition. Across multiple pivotal clinical trials, this class of gene-silencing agents induced either the stabilization or outright reversal of disease progression at the cohort level. Specifically, both neuropathy impairment metrics and neuropathy QoL assessments demonstrated improvement or stabilization relative to patient baselines [146,147,148,149,150,151]. These outcomes underscore the capacity of TTR silencers to deliver a profound therapeutic advantage that transcends mere deceleration of the disease course. A summary of the therapies described is exemplified in Table 1.

## 7. Limitations, Research Gaps, and Future Directions

Although AD is increasingly recognized as a major determinant of morbidity and prognosis in CA, important gaps limit our understanding of its mechanisms and clinical implications. Most available evidence originates from small or heterogeneous cohorts, with scarce use of standardized autonomic endpoints, making the temporal relationship between amyloid burden, vagal–sympathetic imbalance, and neuroinflammation difficult to define.

Disease-modifying therapies have demonstrated clear benefits on neuropathy progression, nutritional status, and quality of life. However, based on currently available evidence, their specific impact on AD remains only partially characterized, as most clinical trials have not incorporated standardized cardiovagal or sympathetic endpoints. Consequently, it is still uncertain to what extent these agents may influence the trajectory of autonomic degeneration.

Major unmet needs include the absence of longitudinal studies exploring how central–peripheral neuroinflammatory pathways and loss of vagal anti-inflammatory signaling contribute to autonomic decline in different amyloid subtypes. Future research should integrate quantitative autonomic testing, neuroinflammatory profiling, and multimodal imaging to elucidate the progression of autonomic impairment and its relationship to arrhythmic risk and survival. Equally important will be the exploration of targeted neuromodulatory or neuroprotective strategies, including vagal modulation, which remain largely unexplored and may open new therapeutic avenues. Moreover, the therapeutic potential of vagal modulation—through non-invasive or targeted neuro-modulatory approaches—remains largely unexplored and may represent a promising direction for translational research.

## 8. Conclusions

The Vagal Link paradigm delineates the crucial structural and functional interplay between the vagus nerve and the myocardium, serving as an integrative mechanism for autonomic homeostasis, intrinsic cardiac neural integrity, and immunomodulatory capacity. Autonomic Dysfunction, particularly vagal efferent impairment, represents a fundamental yet frequently overlooked component of cardiac amyloidosis, linking its cardiac and neurological manifestations. It is also a robust, independent prognosticator of heightened mortality across all disease subtypes. Mechanistically, this impairment is underpinned by a multi-factorial pathological process encompassing direct amyloid deposition, subsequent neurodegeneration, and concomitant neuroinflammation. Clinically, this resulting “vagal phenotype” manifests as a constellation of debilitating symptoms, including orthostatic hypotension, gastrointestinal dysmotility, and cardiac arrhythmias, mandating objective quantification via non-invasive modalities such as HRV analysis. Prospective research endeavors must prioritize the standardization of autonomic endpoints within clinical trials to rigorously evaluate the efficacy of disease-modifying therapies on AD progression, execute longitudinal studies to elucidate the precise contribution of neuroinflammation, establish integrated multimodality diagnostic strategies for comprehensive risk stratification, and explore additional, more specific therapeutic options within this field.

## Figures and Tables

**Table 1:** Summary of therapies and clinical measures addressing autonomic dysfunction and related symptoms in cardiac amyloidosis. Unless otherwise specified, the dose is assumed to be administered via the oral route. ATTR, Transthyretin Amyloidosis; COMPASS-31, Composite Autonomic Symptom Score-31; IV, intravenous; mg, milligram; mmHg, millimeters of mercury; QoL, quality of life; QoL-DN, Quality of Life-Diabetic Neuropathy; SC, subcutaneous; wk, week.

Agent	Dosing	Effects	References
**Non-pharmacological treatment**			
Water	2.0 to 2.5 L per day	↑ Effective intravascular volume	[56]
Salt	1 to 2 teaspoons per day	↑ Effective intravascular volume	[56]
Compression-garment	Exerting a pressure of at least 15–20 mmHg	Optimizing venous return	[119]
**Orthostatic hypotension & vagal tone**			
Fludrocortisone	0.2 mg (loading dose)–0.1 mg/daily	↑ Intravascular volume expansion	[121]
Midodrine	2.5 mg 3 times daily	↑ Systemic vasoconstriction	[3,56,122,125]
Droxidopa	100 mg 3 times daily	↑ Systemic vasoconstriction	[129,130,131]
Pyridostigmine	30 mg 2–3 times daily	Enhancement of cholinergic neurotransmission	[136,137,138]
**Disease-modifying therapy (ATTR)**			
***TTR stabilizers***			
Diflunisal	250 mg 2 times daily	Attenuated the rate of neurological impairment progression	[140]
Tafamidis	20 mg daily 80 mg daily	Slowing the deterioration of sensory-motor peripheral neuropathy	[141,142]
Acoramidis	800 mg twice daily	Insufficient data on autonomic dysfunction at present	Ongoing Clinical trial (NCT06563895)
***TTR silencers***			
Patisiran	0.3 mg/kg every 3 wk, IV	Improved scores on the COMPASS-31 assessment (and other QoL measures) over the placebo group	[147,152]
Vutrisiran	25 mg every 3 mo, SC	Significantly improved multiple measures of QoL (including total Norfolk QOL-DN and the individual autonomic domain)	[146,153]
Inotersen	284 mg once a wk, SC	Stabilization of neuropathy symptoms, including autonomic manifestations.	[148,151]
Eplontersen	45 mg once a month, SC	Sustained positive effects across a diverse range of autonomic impairment metrics.	[149,150]

## Data Availability

No new data were created or analyzed in this study.

## Abbreviations

The following abbreviations are used in this manuscript:

ABPM	Ambulatory Blood Pressure Monitoring
AD	Autonomic Dysfunction
AF	Atrial fibrillation
AL	Immunoglobulin Light-Chain Amyloidosis
ANS	Autonomic Nervous System
ASO	Antisense oligonucleotide
ATTR	Transthyretin Amyloidosis
ATTRv	Hereditary Transthyretin Amyloidosis
ATTRwt	Wild-Type Transthyretin Amyloidosis
AuF	Autonomic Failure
BP	Blood pressure
BRS	Baroreflex sensitivity
CA	Cardiac Amyloidosis
CASS	Composite Autonomic Severity Score
CAP	Cholinergic Anti-Inflammatory Pathway
^11^C-mHED	^11^C-hydroxyephedrine
CMR	Cardiac Magnetic Resonance
CNS	Central Nervous System
CTS	Carpal Tunnel Syndrome
CV	Coefficient of variation
CVR-R	Coefficient of variation in the R-R interval
DPD	3,3-Diphosphono-1,2-propanodicarboxylic acid
ECG	Electrocardiogram
ED	Erectile Dysfunction
GI	Gastrointestinal
GU	Genitourinary
HF	Heart Failure
HFpEF	Heart Failure with Preserved Ejection Fraction
HGS	Handgrip strength
HMDP	Hydroxymethylene diphosphonate
HMR	heart-to-mediastinum ratio
HRDB	Heart Rate Response to Deep Breathing
HRT	Heart Rate Turbulence
HRV	Heart Rate Variability
HUTT	Head-up tilt test
ICNS	Intrinsic cardiac nervous system
IENFs	Intraepidermal nerve fibers
^123^I-MIBG	Iodine-123-metaiodobenzylguanidine
IL	Interleukin
IV	intravenous
LVEF	Left ventricular ejection fraction
MIBG	Metaiodobenzylguanidine
mg	milligram
mmHg	millimeter of mercury
NfL	Neurofilament Light Chain
NSAID	Nonsteroidal anti-inflammatory drug
NTS	Nucleus Tractus Solitarius
OH	Orthostatic Hypotension
PET	Positron emission tomography
PVN	Paraventricular Nucleus
QoL	Quality of Life
QoL-DN	Quality of Life-Diabetic Neuropathy
QSART	Quantitative Sudomotor Axon Reflex Test
RAGE	Receptor for Advanced Glycation End-Products
RMSSD	Root Mean Square of Successive Differences
RNA	Ribonucleic acid
ROS	Reactive Oxygen Species
RVLM	Rostral Ventrolateral Medulla
SBP	systolic blood pressure
SC	subcutaneous
SDRR	Standard deviation of R-R intervals
siRNA	Small interfering RNA
SPECT	Single Photon Emission Computed Tomography
SSR	Sympathetic Skin Response
^9m^Tc-PYP	^99m^technetium-pyrophosphate
THAOS	Transthyretin Amyloidosis Outcomes Survey
TNF-α	Tumor Necrosis Factor Alpha
TST	Thermoregulatory Sweat Test
TTR	Transthyretin Protein
VN	Vagus Nerve
wk	week
WR	washout rate
